# A Combination of Keyhole GTAW with a Trapezoidal Interlayer: A New Insight into Armour Steel Welding

**DOI:** 10.3390/ma12213571

**Published:** 2019-10-31

**Authors:** Zhenyu Fei, Zengxi Pan, Dominic Cuiuri, Huijun Li, Azdiar A. Gazder

**Affiliations:** 1 School of Mechanical, Materials, Mechatronic and Biomedical Engineering, University of Wollongong, Northfield Avenue, Wollongong, NSW 2522, Australia; zf996@uowmail.edu.au (Z.F.); dominic@uow.edu.au (D.C.); huijun@uow.edu.au (H.L.); 2 Defence Materials Technology Centre, 24 Wakefield Street, Hawthorn, VIC 3122, Australia; 3 Electron Microscopy Centre, University of Wollongong, Squires Way, Wollongong, NSW 2500, Australia; azdiar@uow.edu.au

**Keywords:** K-GTAW, armour steel, interlayer, microstructure, low temperature phase transformation, keyhole welding, TRIP

## Abstract

The ballistic performance of armour steel welds using austenitic filler materials is poor on account of the disparity in the mechanical properties of the weld and base metals. Consequently, a novel Keyhole Gas Tungsten Arc Welding process with a trapezoidal AISI309 austenitic stainless steel interlayer was developed to tailor chemical composition and microstructure by controlling the solidification sequence. Results show that the dilution rate in the weld metal region can reach up to 43.5% by placing a specially designed interlayer in between the base metal, providing a major scope for microstructure modification. Detailed weld analysis was undertaken by X-ray diffraction, optical and secondary and transmission electron microscopy, energy dispersive spectroscopy and electron back-scattering diffraction. The results from Vickers hardness indents and Charpy impact toughness testing at −40 °C show that the properties of the weld metal region are comparable to that of the base metal. This is ascribed to the weld metal comprising a two phase microstructure of martensite and retained austenite, which contribute to improvements in strength and toughness, respectively. Furthermore, the tailored chemical composition, microstructure and low temperature phase transformation in the weld metal may reduce the tendency toward both solidification cracking and hydrogen assisted cold cracking.

## 1. Introduction

Military combat vehicles often require good ballistic protection along with weight minimisation in order to maintain high efficiency and performance in extreme environments [1]. Quenched and tempered (Q&T) steels are the most suitable candidates for such application as they possess high hardness with good toughness and a much higher strength to weight ratio than normal carbon steels [2]. It is generally accepted that the harder the material, the better its ballistic performance [3]. Thus, to ensure good ballistic performance, the tempering temperature of armour steels after quenching is relatively low (<200 °C) compared to conventional Q&T steel in order to achieve as high a hardness as possible, while avoiding shattering failure mode. Concurrently, high strength and hard Q&T armour steels also suffer from poor weldability as they are susceptible to hydrogen assisted cold cracking (HICC) [4].

In order to improve the weldability of Q&T steel and produce sound and high quality weldments, austenitic stainless steel (ASS) filler materials are conventionally used, as the solubility of hydrogen in austenitic phase is higher compared to the ferritic phase [5]. However, it has been demonstrated by Reddy et al. [6] that weld metals deposited with ASS filler have very poor ballistic performance because of their low hardness value. This means that ASS filler materials may not be suitable for high strength Q&T steels when ballistic performance is the critical design factor, as reported by Balakrishnan et al. [7]. In an effort to ensure sufficient ballistic performance in the weld metal region of high strength Q&T steels, Balakrishnan et al. [8] proposed the hardfacing technique which involves the deposition of high chromium and high carbon fillers in-between austenitic layers. Although ballistic performance was improved after hardfacing, the inhomogeneous microstructure in the weld metal region may not produce uniform hardness and satisfactory impact toughness at −40 °C, which are key design parameters for armour steel [9]. In addition, the complex deposition procedure, along with expensive filler materials make this technology uneconomical. In principle, the welding parameters are carefully designed in order to ensure that the properties in the weld are uniform and similar to the base metal in order to guarantee both ballistic performance and structural integrity. Thus, it is highly desirable to develop armour steel welds with both improved ballistic performance and mechanical properties in order to satisfy the design criteria.

Recently, a self-designed electrode that tailored a desired microstructure combination was developed in order to meet the design criteria as well as to ensure sufficient ballistic performance. For example, Murthy et al. [10] obtained a fine mixture of carbide free banitic ferrite and austenite in armour steel weld metal through additions of cobalt and by holding the weldment at 350 °C for several hours after welding. Pramanick et al. [11] achieved a bainite and austenite two phase microstructure in the weld metal using a high carbon and high nickel coated electrode. It was reported that both these two welds presented excellent ballistic performance along with mechanical properties comparable to the armour steel base plate. Nonetheless, post-weld heat treatment at 350 °C would decrease the hardness of base metal, while the high carbon high nickel concentration tends to follow a single phase austenitic solidification mode that could in turn be highly vulnerable to solidification cracking on account of the low solubility of impurities in austenite phase. It can be discovered from literature that it is very difficult to achieve the desired combination of chemical composition and microstructure in armour steel weld metal to satisfy both ballistic performance and other design criteria. Since the tempering temperature for armour steel is very low, using post-weld heat treatment to improve the weld properties, and especially hardness and toughness, is not possible. This means that the desired properties of the weld metal need to be obtained in the as-welded condition. This constraint poses a real challenge in the design of an armour steel weld. 

Another problem facing armour steel welding is the low efficiency and productivity associated with the multi-pass fusion welding processes. Recently, Luo et al. [12] applied laser beam welding to weld high strength Q&T steel and thereby increase productivity. Although the hardness of the weld metal was approximately the same as that of the the armour plate, the toughness of weld metal was unsatisfactory due to the formation of untempered martensite. In addition, the poor gap bridging capability and the high capital cost of laser beam welding hinder the development and industrial application of this process considerably. More recently, Fei et al. [13] used Keyhole Gas Tungsten Arc Welding (K-GTAW) to weld armour grade Q&T steel in an attempt to increase productivity. K-GTAW is a novel keyhole welding process similar to laser beam and electron beam welding processes but much cheaper and easier to operate along with much better gap bridging capability. It was demonstrated that up to 12.7 mm thick materials can be welded in a single K-GTAW welding pass [14]. Fei et al. [15] also reported that even though the efficiency of armour steel welding can be improved by the K-GTAW process, it was still very difficult to achieve the desired combination of hardness and toughness in the weld metal region, even with the addition of filler materials. Consequently, it became apparent that appropriate chemical composition and microstructure for armour steel welds produced via the K-GTAW process deserves further in-depth investigation.

In this study, in order to concurrently solve for both metallurgy and efficiency issues, a K-GTAW welding process was used for armour steel welding in combination with an AISI309 austenitic stainless steel interlayer. A specially designed trapezoidal interlayer was introduced for the first time in single pass welding in order to maximize the dilution rate from interlayer materials. Chemical composition and microstructure in the weld metal region were carefully designed by tailoring an appropriate solidification sequence in order to produce a two phase martensite and a retained austenite microstructure. The performance of the armour steel weld was evaluated by both Vickers hardness indents and impact toughness testing at −40 °C. Detailed characterisation of the solidification behaviour and microstructure was undertaken by a combination of Thermo-Calc simulations, and X-ray diffraction (XRD), optical, secondary and transmission electron microscopy, energy dispersive spectroscopy (EDS) and electron back-scattering diffraction (EBSD). Finally, the possible hardening and toughening mechanisms of the armour steel weld, as well as the application potential are presented.

## 2. Materials and Methods 

**Figure 1:**
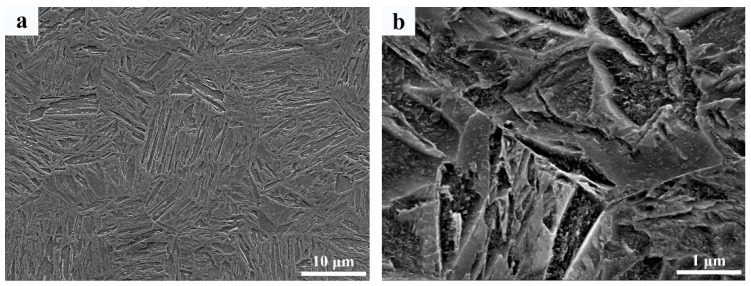
Microstructure of high hardness armour (HHA) base metal; (**a**) low magnification of scanning electron microscope (SEM); and (**b**) high magnification of SEM.

**Figure 2:**
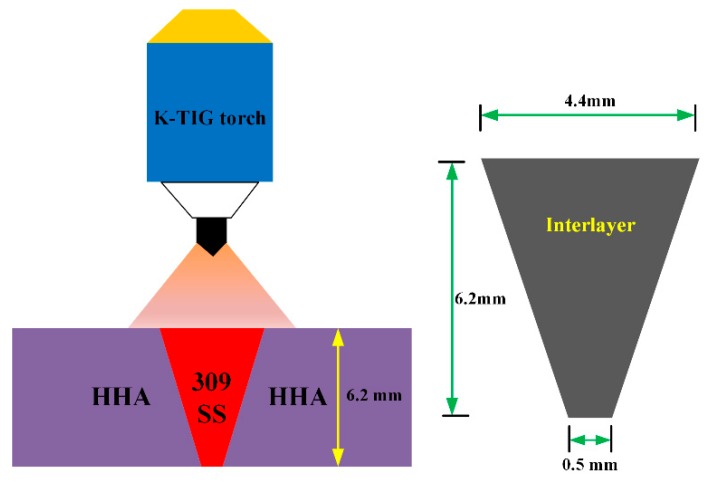
Schematic of the Keyhole Gas Tungsten Arc Welding (K-GTAW) welding process in combination with the interlayer.

The base metal subject to investigation in this study is 6.2 mm thick high hardness armour (HHA) which conforms to the requirement of MIL-DTL-46100 [16]. The interlayer used in-between armour steel plates for welding is AISI309 austenitic stainless steel. The chemical compositions of HHA and 309 interlayer are listed in Table 1. The microstructure of the base metal consists mainly of lath martensite with very fine carbide precipitates, as shown in Figure 1a,b. The K-GTAW welding machine used in this study is composed of a 1000 A power supply, control cabinet, water cooler, specially designed K-GTAW torch, and a control computer used to sense welding current and arc voltage. The AISI309 interlayer with a trapezoid cross section was placed in-between armour steel plates (Figure 2). In order to match the shape of the interlayer and to ensure no gap between the AISI309 interlayer and armour steel, a tailored groove was made on the HHA plate by measuring the base angle of the trapezoid interlayer. A pulsed current waveform was used to conduct the welding process. Detailed welding parameters are listed in Table 2. The surface of the groove as well as the surface of the AISI309 interlayer was cleaned with acetone prior to welding in order to remove all possible contaminants. The AISI309 interlayer was fixed by spot welding at the beginning, middle, and end of the plate. 

**Figure 3:**
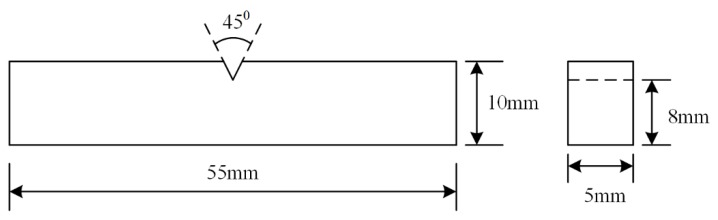
Dimensions of Charpy impact toughness.

After welding, the weld was sectioned for microstructure characterisation and mechanical tests. The sectioned coupon was hot mounted, ground, and polished up to the colloidal silica stage and then etched in a solution of 5 g FeCl_3_, 15 mL HCl, and 60 mL distilled water. Cross-sectional optical macrographs and detailed optical micrographs were captured using a Leica M205A stereoscope (Leica Microsystems Pty Ltd., Sydney, Australia) and a Nikon Eclipse LV100DNA optical microscope (Nikon Corporation, Tokyo, Japan), respectively. EDS and EBSD was conducted on a JEOL JSM-7001F field emission gun-scanning electron SEM microscope (JEOL Ltd., Tokyo, Japan) operating at 15 kV accelerating voltage and ~5.1 nA probe current. For EDS mapping, a 12 mm working distance was used. For EBSD, a 0.15 µm step size was used for the base metal while both 0.15 µm step size and 0.6 µm step size was applied on the weld metal. Post-processing of the EBSD maps was undertaken using the Channel-5 software suite (Oxford Instruments Company, Abingdon, UK). In brief, it involved the removal of wild spikes and cyclic extrapolation of zero solutions up to five neighbours followed by thresholding the band contrast to delineate the unindexed regions. The phase and inverse pole figure (IPF) maps are shown along with the misorientation angle histograms of each phase and the interphase region. In order to characterise fine-scale microstructural features in regions without inter-dendritic retained austenite, electron transparent lamellae were produced via liftout on an FEI Helios Nanolab G3 CX dual beam FIB-SEM (Thermo Fisher Scientific, Hillsboro, OR, USA) and analysed in a JEOL JEM-ARM200F transmission electron microscope (JEOL Ltd., Tokyo, Japan) operating at 200 kV. X-ray diffraction experiments were performed on a Panalytical goniometer (Malvern Panalytical Ltd., Cambridge, UK) using Ni-monochromated Cu Kα radiation (λ = 0.154 nm) at 40 kV and 45 mA in the Bragg-Brentano geometry. The instrument works in a pre-set time mode with sample holder spinning during the whole scanning process in order to compensate for the preferred orientation. The evolution of relative intensity for all samples was measured by varying 2θ from 30° to 140°. Phase volume fraction were estimated through Rietveld refinement by collecting the integrated intensities of (111)γ, (200)γ, (220)γ, and (311)γ peaks of austenite and the (110)α’, (200)α’, (211)α’, and (220)α’ peaks of martensite. Dislocation density in martensite was estimated through the Modified Williamson-Hall (MWH) method. Silicon standard was also scanned with the same instrument setting as experimental samples to account for the instrument broadening. Vickers microhardness indentation was undertaken across the weld in the transverse direction 2 mm below the front surface at 0.5 mm intervals using a 1 kgf load on a Struers DuraScan-70 automatic hardness tester (Struers, Ballerup, Denmark). Charpy impact toughness testing was conducted at −40 °C using subsize specimens in accordance with the ASTM E23 standard (Figure 3). The fracture surface was observed using a JEOL JSM-6490 SEM operating at a 15 kV accelerating voltage, ~5 nA probe current, and 10 mm working distance.

## 3. Results

### 3.1. Macrostructure and Dilution

**Figure 4:**
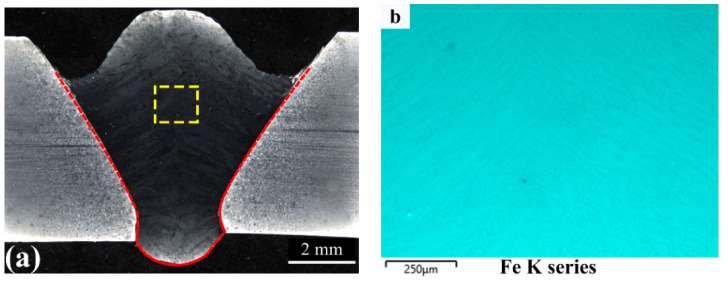

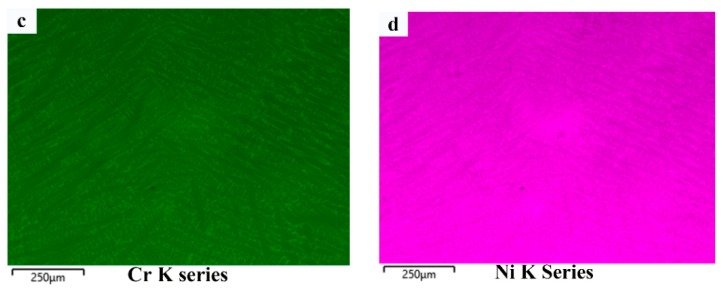
Macrograph and Element distribution map of weld; (**a**) Macrograph of the weld metal; and (**b**–**d**) Element distribution of Fe, Cr, and Ni in weld metal respectively.

The cross-sectional macrostructure of the weld is shown in Figure 4a. Complete fusion between the AISI309 stainless steel interlayer and the HHA base metal was achieved with the K-GTAW welding process. No evident defects, such as porosity, lack of fusion, incomplete penetration, or cracks were found in the weld. However, undercuts were present in the weldment in the front surface. This phenomenon was also observed by Cui et al. [17] during the pulse mode K-GTAW welding process. This might be a result of the stirring effect resulting from pulse mode current waveform, which changes the weld pool fluidity and cooling rate. 

The elemental distribution in the centre region of the weld metal (yellow square) was measured by EDS mapping and is shown in Figure 4b–d. It is shown that both chromium and nickel were enriched in the interdendritic region, suggesting a micro-segregation during solidification. The chemical composition of Ni and Cr was measured by EDS mapping, whereas the concentration of other elements was calculated based on the dilution from the AISI309 stainless steel interlayer. The measured concentration of Cr and Ni in the weld metal were 11.4% and 5.5%, respectively. The average dilution from AISI309 stainless steel was calculated to be 43.54%. Detailed chemical composition was listed in Table 3. It can be seen that with a specially designed interlayer shape, the dilution rate can be ≥40%, which provides the design space necessary for microstructure modification compared to the process involving filler materials, as demonstrated by Fei et al. [15].

### 3.2. Phase Constituents

**Figure 5:**
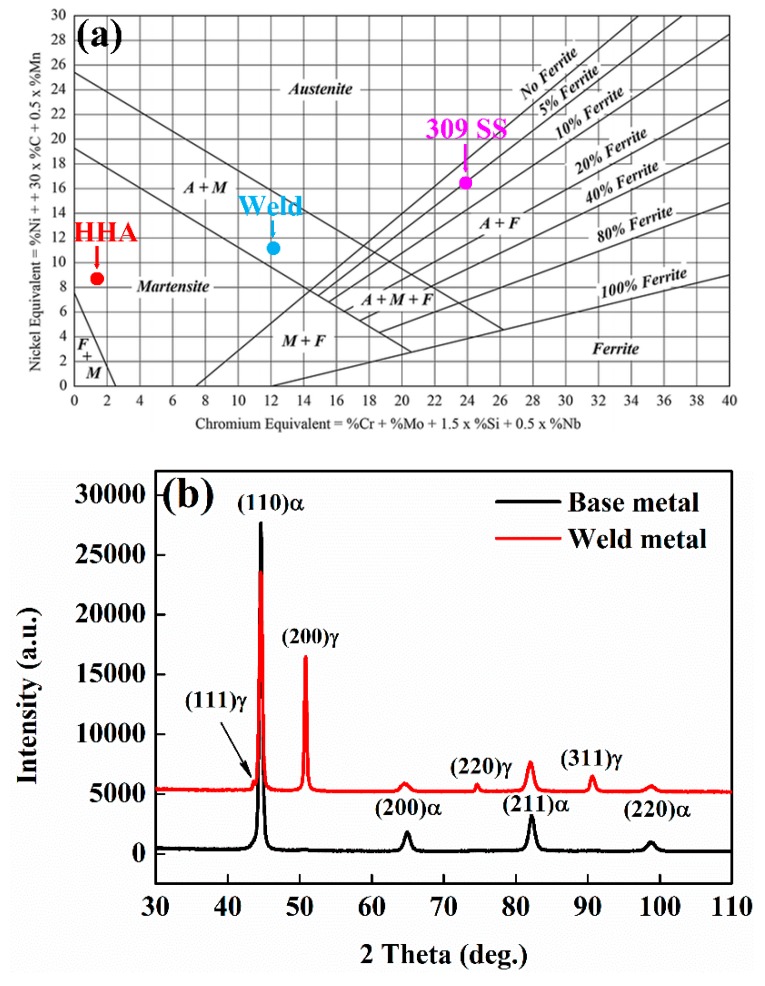
Phase constituents in weld and base metal; (**a**) Schaeffler diagram; and (**b**) XRD patterns.

The phase constituents in the weld metal region can be estimated by the Schaeffler diagram based on Cr and Nickel equivalents. The Cr_eq_ and Ni_eq_ for the weld metal (Cr_eq_ ~ 12.32, Ni_eq_ ~ 11.38) were calculated according to the detailed composition listed in Table 3 and the calculation formula exhibited in Figure 5a. The weld is located in the two phase (martensite and retained austenite) region. The results were further confirmed by XRD (Figure 5b) in which both the bcc and fcc phases were detected. The estimated volume fraction of retained austenite was 24.6% according to Rietveld refinement. Based on the XRD data, only one bcc phased was present in the HHA base metal.

### 3.3. Microstructure

**Figure 6:**
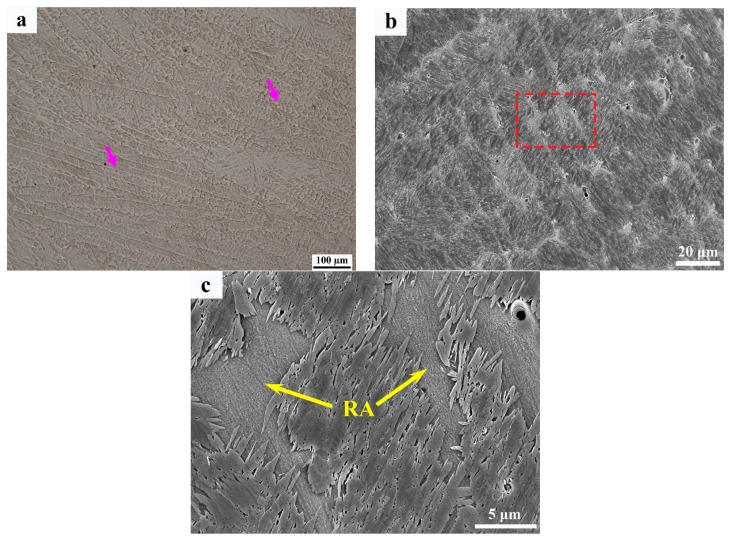
Microstructure of weld metal; (**a**) OM image of weld metal; (**b**) SEM image of weld metal; and (**c**) Magnified SEM image of red square area in (**b**).

**Figure 7:**
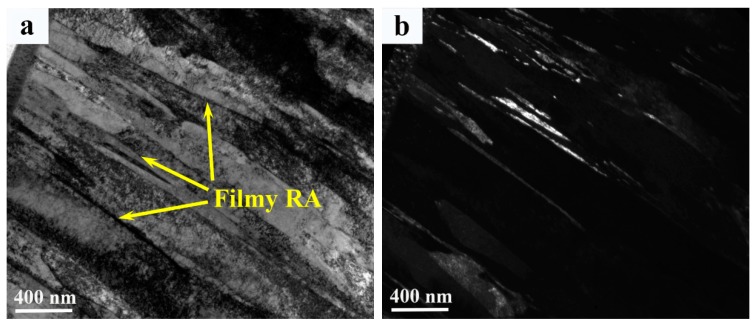

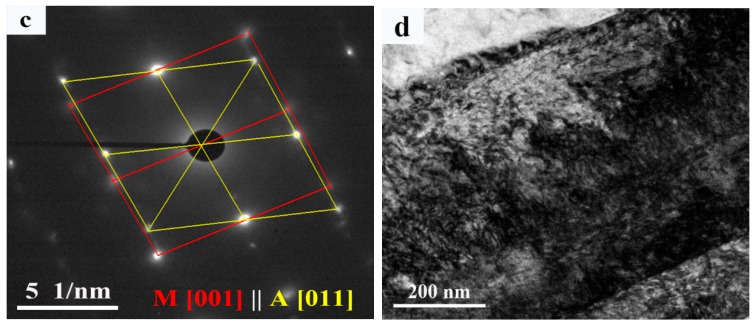
TEM images of weld; (**a**) and (**b**) Bright and dark field image of the weld metal respectively; (**c**) Selective area diffraction pattern of the weld metal; and (**d**) Dislocations inside martensite lath of the weld metal.

The optical and secondary electron micrographs of the weld metal are shown in Figure 6. The microstructures of the weld metal comprises martensite and interdendritic retained austenite as indicated by the purple arrow in Figure 6a. The width of interdendritic retained austenite in the weld metal was ~2–20 µm (Figure 6b,c). An in-depth analysis of interlath filmy retained austenite was carried out by TEM, as shown in Figure 7. The bright-field, dark-filed images and corresponding diffraction patterns (Figure 7a–c) show that filmy retained austenite ≤100 nm in width was detected in between martensitic laths. In addition, highly dislocated substructures were found within martensitic laths (Figure 7d), which is characteristic of martensite transformation. No precipitates were found in the weld metal region.

**Figure 8:**
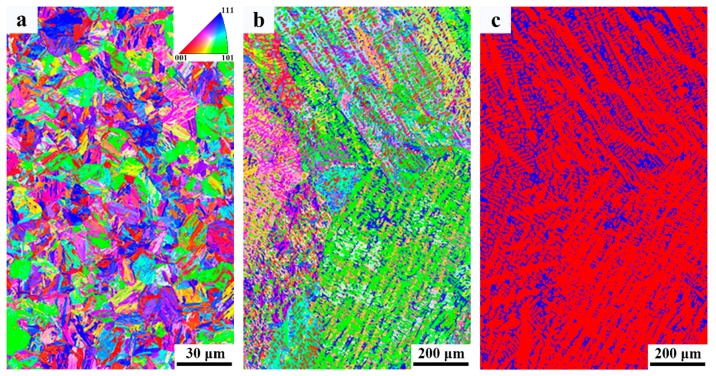
Electron back scattered diffraction (EBSD) maps of both base metal and weld metal; (**a**) and (**b**) Colour inverse pole figure (IPF) for base metal and weld metal, respectively; (**c**) Phase map for weld metal where red and blue represent martensite and austenite, respectively.

**Figure 9:**
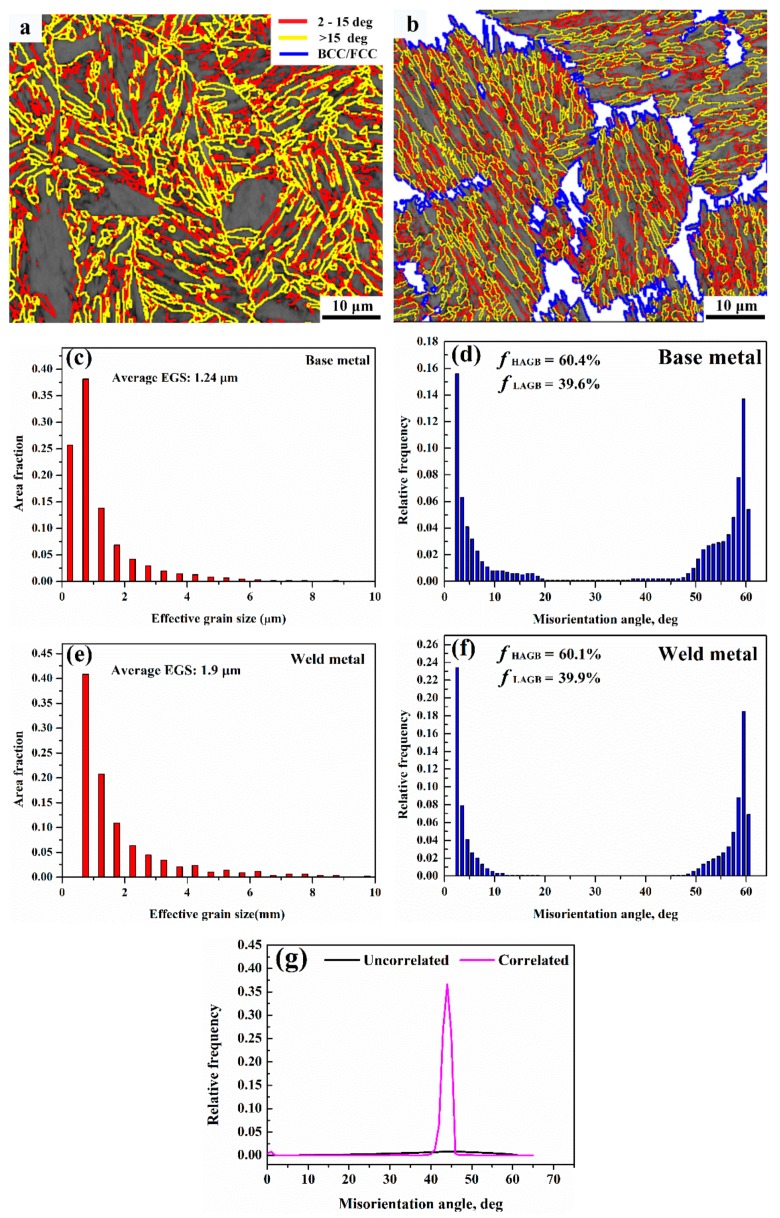
Statistics of effective grain size and misorientation angle; (**a**) and (**b**) Misorientation distribution maps superimposed on band contrast map in a BCC structure for base metal and weld metal, respectively; (**c**) and (**d**) Effective grain size for base metal and weld metal respectively; (**e**) and (**f**) Misorientation angle distribution for base metal and weld metal, respectively; (**g**) Misorientation angle distribution between martensite and austenite in the weld metal.

Phase band contrast and IPF maps, and phase map are shown in Figure 8. Figure 8a,b depicts similar orientations in martensitic laths forming blocks, followed by the coalescence of the blocks into packets with different lattice planes. From phase map (Figure 8c) it is evident that the absolute majority of retained austenite is located in the interdendritic region. The fraction of retained austenite estimated from EBSD phase map is 21%, which is consistent with the XRD results. The smaller fraction detected by EBSD is probably a result of a large step size used (0.6 µm), which makes it impossible to detect smaller retained austenite. The misorientation distribution map of martensite is shown in Figure 9a,b. As shown by Long et al. [18], boundary misorientation <15° denote laths, whereas boundaries between 15° to 45°, 45° to 55°, and 55° to 62.8° denote prior austenite, packet, and block boundaries, respectively. Figure 9c–f shows the quantitative statistics of effective grain size and misorientation distribution. The 1.24 µm average effective grain size of the base metal is smaller than that of the weld metal (1.9 µm). The fraction of high-angle grain boundaries of the base and weld metal are similar. The misorietation between martensite and retained austenite is also large angle boundaries (~45°), as shown in Figure 9g.

### 3.4. Hardness

**Figure 10:**
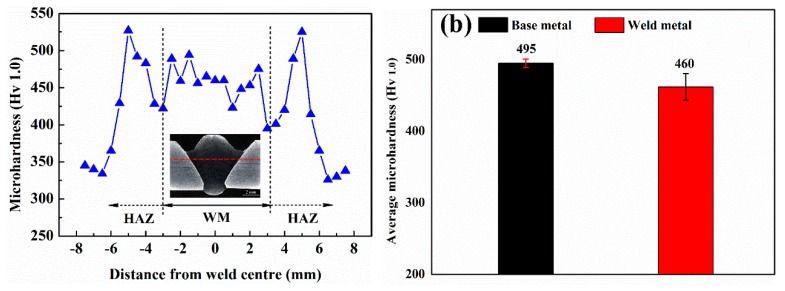
Hardness results; (**a**) Hardness distribution across weld; and (**b**) Average hardness for base metal and weld metal.

The microhardness across the weld is shown in Figure 10. It can be seen that the average hardness in the weld metal region is much higher than that obtained using both austenitic (~250 Hv) and ferritic (~300 Hv) filler materials. Furthermore, the hardness of the weld is similar to that obtained using self-designed matching filler materials and is very close to the hardness range (470 –550 Hv) specified for 500 grade armour steel plate base metal.

### 3.5. Impact Toughness

**Figure 11:**
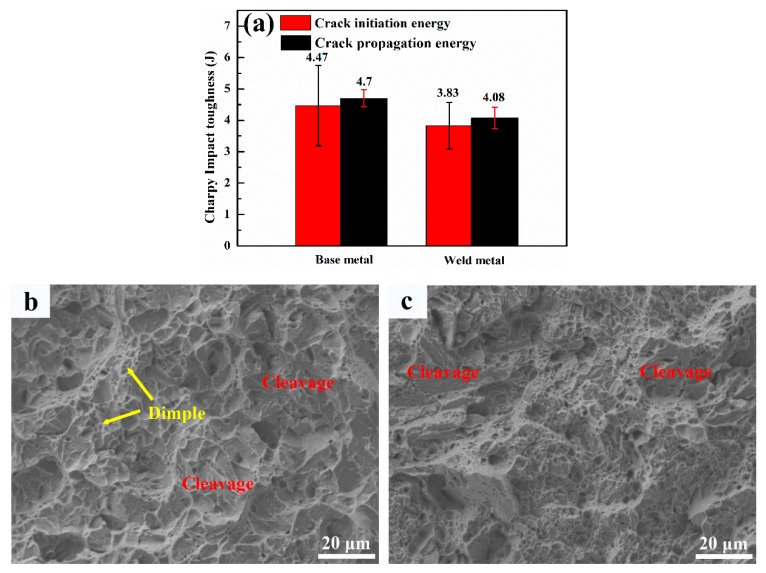
Charpy impact toughness results; (**a**) Charpy impact toughness value for base metal and weld metal; and (**b**) and (**c**) SEM images of fracture surface of base metal and weld metal, respectively.

The results of impact toughness testing at −40 °C is shown in Figure 11a. The impact toughness of the weld metal is 7.91 J, which is comparable to the base metal (9.17 J). The base metal exhibits quasi-cleavage fracture mode with predominant river patterns inside cleavage facets, along with small fraction of dimples (Figure 11b). Similarly, a local cleavage fracture was also observed in the weld metal fracture surface (Figure 11c). However, the area fraction of the cleavage fracture was smaller.

## 4. Discussion

### 4.1. Solidification Sequence

**Figure 12:**
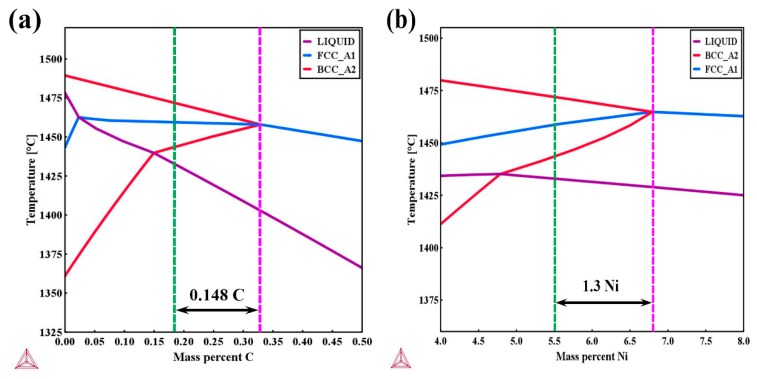
Thermo-calc calculation of solidification sequence of weld; (**a**) and (**b**) Solidification sequence of weld as a function of carbon and nickel content respectively.

The equilibrium phase diagram was calculated by Thermo-Calc software based on the detailed chemical composition in the weld metal. The green and purple dotted lines represent the solidification sequence of the investigated weld and the critical transition point for a single austenitic phase solidification mode, respectively. It can be seen from Figure 12 that by carefully tailoring the ratio of Cr_eq_ to Ni_eq_ the weld solidified from liquid to delta ferrite first, followed by the peritectic reaction from liquid + delta ferrite to austenite. On one hand, the solubility of sulphur and phosphorous in delta ferrite is much higher than that in austenite, which dramatically reduces the enrichment of these harmful impurities in the final liquid compared to single austenitic solidification mode in high carbon high nickel welds and suppresses the initiation of solidification cracking. On the other hand, for welds solidifying in the primary ferritic mode, the solidification grain boundaries can be eliminated by a three-phase reaction during solidification, which results in more irregular austenite/delta ferrite boundaries serving as crack attesters [19]. Thus, the tendency towards solidification cracking for the investigated weld becomes very low, which is beneficial for structural integrity. It is worth mentioning that the coefficient for conversion from carbon content to nickel equivalent is 30 based on a Schaeffler diagram. Thus, as can be discovered from Figure 12a,b, compared to the addition of nickel, the addition of carbon contributes much more to nickel equivalent and therefore has a higher tendency to stabilize austenite without falling into the single phase austenitic solidification mode. In other words, the addition of carbon and reduction in nickel content have the potential of increasing retained austenite content at room temperature, which would provide a promising method towards improving the design of armour steel welds.

### 4.2. Microstructure Evolution

The microstructure in the weld metal is linked to the chemical composition and cooling rate. Due to the addition of a large amount of alloying elements, such as Cr, Ni, and Mn, the hardenability of the weld metal is sufficient to form martensite during air cooling, which is in good agreement with the Schaeffler diagram. Considering that the individual effects of various alloying elements are hard to identify, the microstructure is analysed using its overall chemical composition. The martensite transformation start temperature (Ms) can be predicted by the following empirical equation [20] as listed below:

(1) Ms (℃)=561−474×(C)−33×(Mn)−17×(Ni)−17×(Cr)−21×(Mo)

**Figure 13:**
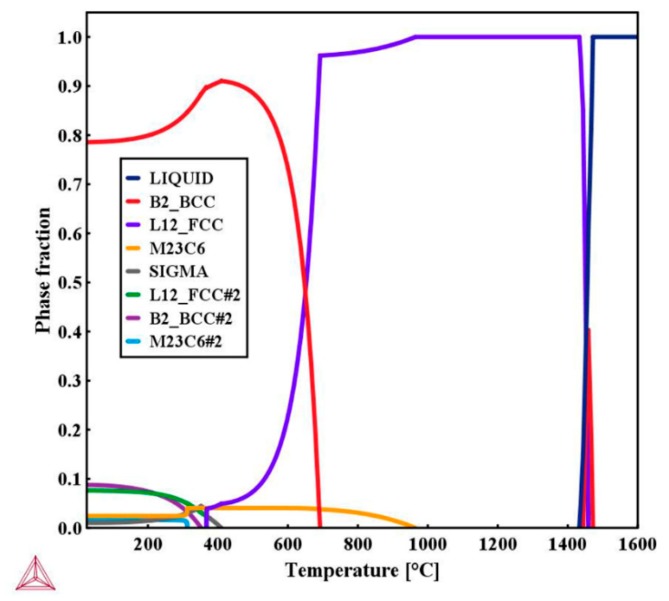
Calculation of the equilibrium phase diagram of weld metal.

The calculated Ms for the weld metal is 158.3 °C. As Ms decreases, the phase transformation from austenite to martesnite may become incomplete due to the simultaneous decrease in the martensite transformation finish temperature (Mf). Normally, the difference between Ms and Mf is around 200 °C. Thus, a certain amount of austenite remains untransformed at room temperature. In addition, alloying enrichment occurs in the interdendritic region during solidification, such as for Cr and Ni as shown in Figure 4, indicating that the Ms in the interdendritic region will be lower than 158.3 °C while that in the dendritic core is higher than 158.3 °C. That is why a majority of the retained austenite was distributed in the interdendritic region. The equilibrium phase diagram of the weld calculated by the Thermo-Calc software is shown in Figure 13. Although precipitates such as M_23_C_6_ and Sigma phase were predicted in the equilibrium phase diagram, no precipitates were found in the weld. This is probably the result of the fast cooling rate during welding as well as the relatively lower rates of diffusion of alloying elements in the fcc phase. Similar results were also reported by Song et al. [21] in Fe-Cr-Ni-Mo martensitic stainless steel.

### 4.3. Hardening Mechanism

There are five main strengthening mechanisms in materials, namely solid solution, dislocation density, phase balance, grain size, and precipitation strengthening. First of all, since Cr, Ni, and Mn are substitutional solid solution elements, they can generate local non-uniformity in the crystal lattice and local stress fields, which makes it more difficult for plastic deformation to occur by hindering dislocation movement and in turn results in an increase in hardness in the weld region. Secondly, the dislocation density of the base and weld metal estimated from XRD pattern are 3.6 × 10^15^ m^−2^ and 2.2 × 10^16^ m^−2^, respectively. Thus, the higher dislocation density in the weld metal would provide additional hardening effects [22]. The higher dislocation density in the weld metal is probably a result of low temperature phase transformation, which allows less space for stain accommodation. It is worth mentioning that even though the dislocation density calculated by the MWH method might be slightly overestimated, as reported by Takebayashi et al. [23], it will not affect the parallel comparison between the base and weld metal. Although solid solution strengthening and dislocation strengthening favour high hardness for the weld metal, the presence of soft retained austenite and larger grain sizes are the possible reasons for the lower hardness of the weld compared to the HHA base metal. As the grain size becomes larger, the number of grain boundaries decrease, with fewer barriers pinning dislocation movement and thereby reducing hardness. It is worth noting that carbon is a critical element determining the hardness in martensite [24]. This is another reason for the lower hardness in the weld (0.183% C) compared to the HHA base metal (0.27% C).

### 4.4. Toughening Mechanism

**Figure 14:**
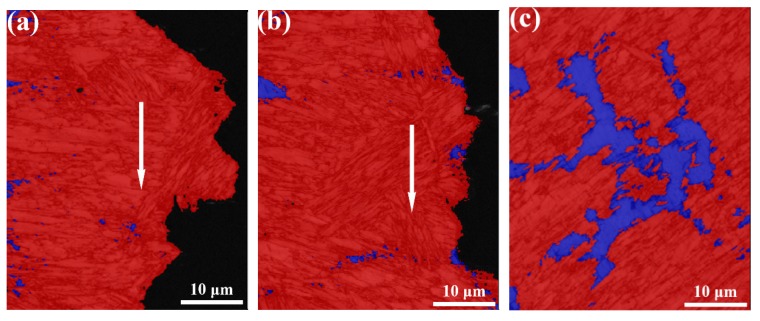
Phase map superimposed on band contrast map; (**a**) Crack initiation area in the weld metal; (**b**) Crack propagation area in the weld metal; (**c**) Deformation-free area in the weld metal. Note: Red and blue represent martensite and austenite, respectively. The white arrow represents the crack path.

It is accepted that toughness is related to several factors, such as dislocation density or stress concentration, microstructure and grain size [25]. As reported by Zhou et al. [26], higher dislocation density or stress concentration is deleterious to toughness as dislocation clusters tends to act as initiation sites for microcracks. In addition, the fracture stress is closely linked to the effective grain size [27], as shown in the equation below:

(2) $$\sigma f={\left[\left(4E\gamma \rho \right)/\left(1-v^{2}d\right)\right]}^{1/2}$$

where σf is fracture stress, E is the Young’s modulus, v is Poisson’s ratio, γp is plastic deformation energy, and d is the effective grain size (EGS). It can be seen from Equation (2) that the fracture stress decreases as EGS increases, resulting in lower deformation before crack initiates. Thus, the higher dislocation density and larger grain size in the weld metal would lower the crack initiation energy. However, the crack initiation energy of the weld metal is similar to the base metal (Figure 11a), which implies that retained austenite plays an important role in enhancing toughness during the crack initiation stage. As reported by Ma et al. [28], retained austenite is more ductile than martensite and will deformed first via the so-called DARA (dislocation absorbed by retained austenite) effect [29]. Martensite being the harder phase remains intact at the start of deformation. Once the critical stress for phase transformation from retained austenite to martensite is reached, the volume expansion resulting from the transformation induced plasticity (TRIP) effect can absorb large amounts of energy during fracture [30]. This phenomenon is reflected along the tortured fracture path, as shown in Figure 14a. The amount of retained austenite adjacent to the crack initiation area is much less than that in the deformation-free area (Figure 14c). It is evident that both DARA and TRIP effects compensate for the loss of crack initiation energy associated with the higher dislocation density and larger grain sizes in the weld metal. 

**Figure 15:**
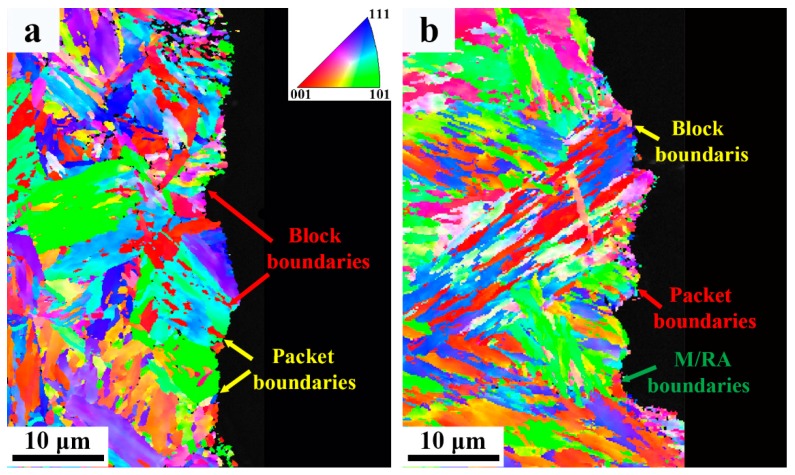
EBSD IPF maps showing the fracture path; (**a**) Base metal; and (**b**) weld metal.

**Figure 16:**
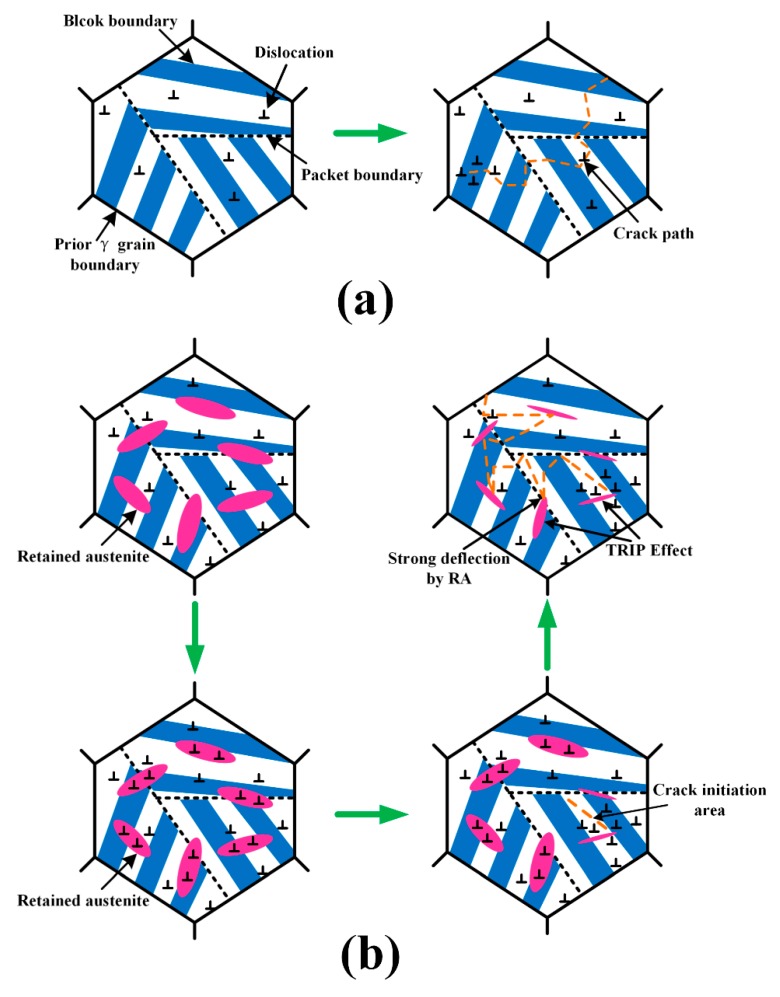
Schematic of the fracture path; (**a**) HHA base metal; and (**b**) weld.

Once a crack forms, subsequent crack propagation is governed mainly by high angle grain boundaries [31]. It was reported by Hu et al. [32] that high-angle grain boundaries are very effective in deflecting or even arresting crack propagation while low-angle grain boundaries never produce significant deflection or arrest of cracks. As shown in Figure 9, the fraction of large angle grain boundaries of the weld and base metals (>15°) is similar, whereas the grain size of the base metal is finer compared to the weld metal. Therefore, the smaller EGS means a greater number of high-angle grain boundaries, which introduce more crack deflection and consume more energy during crack propagation. However, the boundaries between retained austenite and marteniste is also large angle grain boundaries, which may compensate for the less amount of total effective boundaries to some extent. Similarly, the TRIP effect also comes into play during the crack propagation process, as shown in Figure 14b. In addition, it was also reported that retained austenite has the ability to cause large angle twists when cracks encounter the phase [27]. This mechanism was reinforced in Figure 15. It is known that martensite can be divided into several packets, which are further divided into blocks and laths. Since both packet boundaries and block boundaries are large angle grain boundaries, they both can act as effective barriers for crack propagation in the base metal, whereas both block and packet boundaries and retained austenite deflect cracks in the weld metal. The large angle twist from retained austenite is more remarkable than packet and block boundaries and is able to prolong the crack path and consume additional energy. A detailed schematic of the fracture path for both base and weld metals is shown in Figure 16. Therefore, it can be inferred that both the TRIP effect and crack deflection by retained austenite have very positive effects on the increase in crack propagation energy.

### 4.5. Application Potential

As presented above, the desired combination of hardness and toughness of armour steel weld metal similar to base metal was achieved by combination of K-GTAW with an AISI309 stainless steel interlayer, which would also lead to excellent ballistic performance based on the results reported in literature [11]. Moreover, it has already been demonstrated that the tendency towards solidification cracking is very low with a tailored ratio of Cr_eq_ and Ni_eq_. However, it is worth noting that in order for the weldment to be used in real fabrication processes, other factors also need to be taken into account, such as HICC, which is characteristic of high strength Q&T steel. It is well known that the three factors that affect hydrogen assisted cold cracking are level of diffusible hydrogen, tensile residual stress and susceptible microstructure [5]. As reported by Kuzmikova [16], if the martensite transformation in weld metal takes place after surrounding the parental metal, the supersaturated hydrogen in the HAZ will diffuse towards the weld metal, which is still austenitic. This is the case in the current study as the alloying elements in the weld metal dramatically lower the Ms. While considering the higher solubility of hydrogen in an austenitic phase, the large fraction of retained austenite (24.6%) at room temperature in the weld metal could reduce dramatically the tendency towards HICC compared with fully martensitic microstructure. In addition, the yield strength of the two phase (martensite and retained austenite microstructure) would be much lower than single phase martensitic microstructure, as reported by Wu et al. [33], which is another benefit of a reduced tendency towards HICC. Finally, as the Ms in the weld is low (158.3 °C), the volume expansion induced by phase transformation from austenite to martensite could significantly reduce the tensile residual stress in the weld metal region [34]. Therefore, this kind of chemical composition and microstructure is considered as a very promising option for armour steel welding. Future research will focus on the effect of carbon concentration and austenite fraction on HICC, fatigue properties, and ballistic performance.

## 5. Conclusions

Single pass full penetration was achieved on a 6.2 mm armour steel joint using K-GTAW welding technology in combination with The AISI309 stainless steel interlayer. The main conclusions are drawn as follow:

1. Through the introduction of specially designed trapezoid interlayer in between HHA, up to a 43.5% dilution rate can be achieved, providing a very great scope for microstructure modification in the K-GTAW welding.
2. By carefully tailoring the ratio of Cr_eq_ to Ni_eq_ in the weld metal, favourable solidification sequence was obtained. The weld solidifies from liquid to delta ferrite first, followed by peritectic reaction from liquid plus delta ferrite to austenite, which has a very low risk of solidification cracking compared to the single austenitic solidification mode due to the reduction of enrichment of S and P impurities in the final liquid suppressing crack initiation and the formation of more irregular austenite/delta ferrite boundaries hindering crack propagation.
3. The microstructure in the weld metal region contains martensite and retained a austenite dual phase because of the addition of large amounts of alloy elements, which lowers martensite transformation finish temperature to a level much lower than room temperature. No precipitates were detected in the weld metal due to the fast cooling rate and low rates of diffusion of alloying elements in the austenitic phase.
4. The hardness of the weld metal reaches up to 460 Hv. Toughness of the weld is comparable to the base metal. The high dislocation density and solid solution provide sufficient strengthening in the weld, while DARA, TRIP effects, and crack deflection by retained austenite are the main reason for the toughness improvement in the weld metal.
5. Due to the mitigation of the three main factors affecting HICC, this kind of chemical composition and microstructure combination shows great potential to produce high performance armour steel joints, while the K-GTAW welding technology provides an advanced solution for armour steel welding at the same time.

## Figures and Tables

**Table 1:** Chemical composition of armour steel and 309 interlayer (wt. %).

Materials	C	Si	Mn	P	S	Ni	Cr	Mo	Fe
HHA	0.27	0.3	0.3	0.014	0.0025	0.19	1.05	0.25	Bal.
3 09	0.07	0.8	1.4	0.02	0.005	13.4	22.7	N/A	Bal.

**Table 2:** Fixed welding parameters.

Process Parameters	Details
Peak current, A	550
Base current, A	230
Duty cycle	50%
Pulse frequency, Hz	5
Travel speed, mm/min	350
Shielding gas	Pure argon
Shielding gas flow rate, L/min	25
Back purging gas	Pure argon
Purging gas flow rate, L/min	5
Arc length, mm	1
Operation mode	DCEN

**Table 3:** Chemical composition of weld metal (wt.%).

Element	C	Si	Mn	P	S	Ni	Cr	Mo	Fe	Dilution (%)
wt%	0.183	0.52	0.78	0.0166	0.0036	5.5	11.4	0.141	Bal.	43.54
